# Obesity Impairs Embryonic Myogenesis by Enhancing BMP Signaling within the Dermomyotome

**DOI:** 10.1002/advs.202102157

**Published:** 2021-10-14

**Authors:** Liang Zhao, Nathan C. Law, Noe A. Gomez, Junseok Son, Yao Gao, Xiangdong Liu, Jeanene M. de Avila, Mei‐Jun Zhu, Min Du

**Affiliations:** ^1^ Nutrigenomics and Growth Biology Laboratory Department of Animal Sciences and School of Molecular Bioscience Washington State University Pullman WA 99164 USA; ^2^ Department of Animal Sciences Washington State University Pullman WA 99164 USA; ^3^ Center for Reproductive Biology College of Veterinary Medicine Washington State University Pullman WA 99164 USA; ^4^ School of Food Science Washington State University Pullman WA 99164 USA

**Keywords:** bone morphogenetic proteins signaling, embryonic myogenesis, maternal obesity, single cell RNA sequencing

## Abstract

Obesity during pregnancy leads to adverse health outcomes in offspring. However, the initial effects of maternal obesity (MO) on embryonic organogenesis have yet to be thoroughly examined. Using unbiased single‐cell transcriptomic analyses (scRNA‐seq), the effects of MO on the myogenic process is investigated in embryonic day 9.5 (E9.5) mouse embryos. The results suggest that MO induces systematic hypoxia, which is correlated with enhanced BMP signaling and impairs skeletal muscle differentiation within the dermomyotome (DM). The Notch‐signaling effectors, HES1 and HEY1, which also act down‐stream of BMP signaling, suppress myogenic differentiation through transcriptionally repressing the important myogenic regulator MEF2C. Moreover, the major hypoxia effector, HIF1A, enhances expression of HES1 and HEY1 and blocks myogenic differentiation in vitro. In summary, this data demonstrate that MO induces hypoxia and impairs myogenic differentiation by up‐regulating BMP signaling within the DM, which may account for the disruptions of skeletal muscle development and function in progeny.

## Graphical abstract

Molecular mechanisms controlling embryonic myogenesis are examined by a pseudo temporal model at the single‐cell level. Moreover, maternal obesity attenuates myogenic differentiation in E9.5 embryos by enhancing bone morphogenetic protein (BMP) signaling within the dermomyotome, which suppresses Mef2c expression. Disrupted embryonic myogenesis provides a mechanistic explanation for the impairments in muscle function of offspring due to maternal obesity.

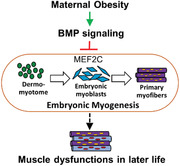

## 1 Introduction

Accompanied with the global obesity epidemic, the prevalence of pre‐pregnancy obesity has increased rapidly over the last few decades.^[^ ^1^ ^]^ According to recent reports, 50 to 60% of women of reproductive age in the United States were pre‐pregnancy overweight or obese.^[^ ^1^, ^2^ ^]^ Maternal obesity (MO) not only induces immediate pregnancy complications such as gestational diabetes and congenital fetal malformations, but also leads to long‐term impairments to the offspring with increased risks of developing metabolic syndromes and neurodevelopmental disorders.^[^ ^1^, ^3^, ^4^ ^]^ Though the underlying mechanisms through which MO induces long‐term impairments in the offspring remain poorly understood, nutritional stress during pregnancy has demonstrated effects on fetal development with long‐term consequences on offspring health, termed as the developmental origins of adult diseases.^[^ ^5^ ^]^ Therefore, previous studies have been extensively focused on the perinatal development affected by MO.^[^ ^4^, ^6^, ^7^, ^8^, ^9^ ^]^ However, tissues and organs formed during the fetal stage are derived from precursors formed during organogenesis at the embryonic stage, and the effects of MO during this initial stage of embryonic organogenesis remains largely unexplored.

MO impairs skeletal muscle development during both fetal and postnatal stages.^[^ ^10^, ^11^, ^12^, ^13^ ^]^ In nonhuman primates, MO impairs oxidative capacity, reduces mitochondrial efficiency, and increases oxidative stress in fetal skeletal muscle.^[^ ^10^ ^]^ Insulin sensitivity is decreased in the skeletal muscle of both fetuses and offspring born to over‐weight or obese mothers.^[^ ^10^, ^11^, ^12^ ^]^ In addition, maternal over‐nutrition induces chronic hypoxia and inflammation, which correlates with impaired fetal myogenesis.^[^ ^13^ ^]^ These adverse changes of fetal muscle due to MO suggest an earlier developmental origin, likely during the embryonic stage.

During embryonic development, the first wave of myogenesis occurs within the dermomyotome (DM), which derives from somites along with the sclerotome (SCL).^[^ ^14^ ^]^ While progenitors within the SCL develop into the vertebral and rib cartilage, myogenic progenitors (MPs) derived from the DM give rise to most of the skeletal musculature, with the exception of craniofacial muscles.^[^ ^15^ ^]^ The myogenic process within the DM is well‐controlled by a group of myogenic regulatory factors (MRFs) under the guidance of signals from surrounding tissues. Pax3 is expressed in the DM at the initial stage of myogenesis, which initiates the expression of Myf5 and myogenic commitment.^[^ ^16^ ^]^ These PAX3+/MYF5+ MPs delaminate from the DM and migrate underneath to form a layer of myotome, where they proliferate and differentiate into embryonic myocytes.^[^ ^15^, ^17^ ^]^ Then, these mononucleated myocytes align along the anterior–posterior body axis and elongate to span the entire somite. Finally, primary myofibers (PMs) are formed by fusion and serve as scaffolds for the formation of secondary muscle fibers during the fetal stage.^[^ ^15^, ^18^ ^]^ Signals of Wnts, Sonic hedgehog (Shh), and bone morphogenetic proteins (BMPs) from surrounding tissues of the DM, such as the dorsal ectoderm, neural tubes, notochord (NOTO), and lateral plates, coordinately regulate the specification and differentiation of the DM.^[^ ^19^, ^20^, ^21^ ^]^ To date, the effects of MO on the embryonic myogenic process remain unexamined.

To better understand the effects of MO on embryonic myogenesis, we profiled the single‐cell transcriptome of mouse embryos at embryonic day 9.5 (E9.5) from lean and obese mothers. A complete myogenic trajectory within the DM was constructed to elucidate the molecular mechanisms underlying myogenic regulation. In addition, we also explored hypoxia and up‐regulation of BMP signaling as potential drivers of impaired embryonic myogenesis due to MO. Stage‐specific impairments to myogenic lineages within the DM caused by MO suggest long‐term disruptions to the development and function of skeletal muscle.

## 2 Results

### 2.1 Single‐Cell Transcriptomic Profiling of E9.5 Embryos

**Figure 1:**
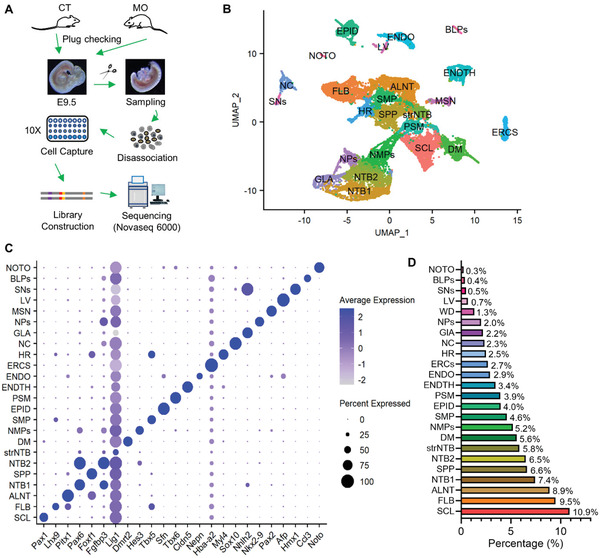
Single‐cell transcriptomic profiling of mouse embryos at embryonic 9.5 day (E9.5). A) Schematic of the experimental pipeline. After mating and plug‐checking, E9.5 embryos were collected from the control or obese (CT and MO) mothers. After disassociation, cell suspensions were processed with the 10X Genomics platform to capture single cells for library construction and sequencing using Illumina Novaseq 6000 (S4). B) UMAP visualization of 24 transcriptionally distinct clusters from integrated dataset of CT and MO samples. Cells with similar transcriptional profiles were grouped in each cluster and were shown in different colors. C) Dot plot showing one selected lineage‐specific marker gene expression for each cluster. D) Overall proportions of each cluster in E9.5 embryos. SCL, sclerotome; FLB, forelimb bud; ALNT, allantois; NTB1, neural tubes 1, SPP, splanchnopleure; NTB2, neural tubes 2; strNTB, stressed neural tubes; DM, dermomyotome; NMPs, neuromesodermal progenitors; SMP, somatopleure; EPID, epidermis; PSM, pre‐somitic mesoderm; ENDTH, endothelium; ENDO, endoderm; ERCS, erythroid cells; HR, heart; NC, neural crest; GLA, ganglia; NPs, Neural Crest; MSN, mesonephroi; LV, liver bud; SNs, sensory neurons; BLPs, blood progenitors; NOTO, notochord.

Mouse embryos at E9.5 were collected from control (CT) and MO groups and digested into single‐cell suspensions for scRNA‐seq analysis (**Figure** **1**A). Before mating, female mice were fed either with a high‐fat diet (HFD) or a control fat diet (CD) for 10 weeks (Figure S1A, Supporting Information). Female mice with HFD‐feeding gained 23 ± 1.5% more body weight than the CT group. HFD‐feeding also elevated fasting glucose and insulin levels with increased HOMA‐IR and impaired glucose tolerance (Figure S1B–E, Supporting Information). At E9.5, after removing the head and the first 7 somites, embryos were disassociated into single cell suspension, captured by a 10× microfluidic chip for the library construction, and sequenced on an Illumina Novaseq6000 S4 flow cells (Figure 1A). After filtering out low‐quality cells, 25 021 single‐cell transcriptomes with a median of 5768 genes and 33 181 unique molecular identifiers (UMIs) per cell were retained for further analysis.

Using unsupervised clustering, integrated cell samples were divided into 24 distinct clusters (Figure 1B) with cell identities determined by canonical marker gene distribution (Figure 1C and Table S1, Supporting Information). Collected single cells from CT and MO groups showed consistent distribution within each cluster (Figure 1D and Figure S1F,G, Supporting Information), suggesting an unbiased capture of cell populations between treatment groups. Clusters were annotated for major cell populations involved in early organogenesis, including the neuromesodermal progenitors, presomitic mesoderm (PSM), SCL, and DM. While a portion of those identified lineage‐specific genes are canonical markers for clustering (Figure 1C and Table S1, Supporting Information), other genes are novel and with functions that remain largely unexplored. For example, the restricted expression of Brachyury (T), Hes7, and Tbx6 in the PSM were previously reported with lineage‐specific regulatory functions.^[^ ^22^, ^23^, ^24^ ^]^ On the other hand, genes such as Ifitm1 and Cited1 were highly enriched (> 28‐fold) within the PSM, but their functions on the regulation of the PSM remain unclear.

### 2.2 MO Impairs E9.5 Embryonic Development

**Figure 2:**
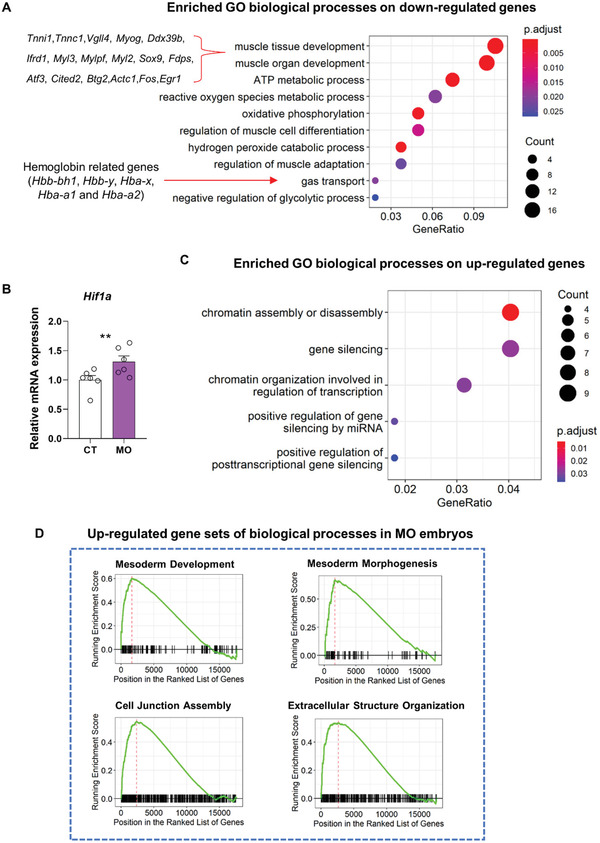
MO induces transcriptomic changes in E9.5 embryos. A) Representative terms of biological processes that are enriched by GO term analysis on down‐regulated genes of the whole cell population in the MO group. B) Relative mRNA expression of Hif1a in E9.5 mouse embryos. Three embryos from each litter were pooled together for mRNA extraction and the litter was an experimental unit. *N* = 6. **p < 0.01 (two tailed *t*‐test). CT, the control group. MO, the maternal obese group. C) Representative terms of biological processes that are enriched by GO term analysis on upregulated genes of the whole cell population in the MO group. D) Representative terms of biological processes that are enriched by GSEA on the whole cell population in the MO group.

Over‐representation analysis (ORA) and gene set enrichment analysis (GSEA) were performed for genes differentially expressed between MO and CT groups (**Figure** **2**A,C,D and Tables S2–S4, Supporting Information). Strikingly, the GO term of “gas transport” was enriched in the down‐regulated genes of MO embryos accompanied with reduced expression of oxygen transporters, the hemoglobin‐related genes (Hbb‐bh1, Hbb‐y, Hba‐x, Hba‐a1, and Hba‐a2) (Figure 2A). Enhanced expression of a major hypoxia effector, hypoxia‐inducible factor 1 *α* (Hif1a) was also detected, which we verified by the RT‐qPCR of E9.5 embryos (Figure 2B). Therefore, these data suggest a systemic hypoxic state in the MO embryos. Consistent to hypoxia‐induced outcomes, biological processes of “oxidative phosphorylation”, “ATP metabolic process”, and “negative regulation of glycolytic process” were all down‐regulated (Figure 2A), suggesting that MO induced a metabolic shift from oxidative phosphorylation to glycolysis in E9.5 embryos. In addition, MO also aggregated oxidative stress in E9.5 embryos by down‐regulating genes related to the biological processes of “reactive oxygen species metabolic process” and “hydrogen peroxide catabolic process”. Consistent with disrupted metabolic processes identified by the ORA, multiple gene sets related to the catabolic processing of phenolic compounds, carboxylic acids, and ketones were down‐regulated in the MO group (Figure S2A, Supporting Information). In addition, multiple GO terms related to gene expression and epigenetic changes such as “gene silencing” and “chromatin organization involved in regulation of transcription” were enriched in the upregulated genes of MO offspring, which is considered as one of the contributing factors to long‐terms effects of MO on offspring (Figure 2C and Table S3, Supporting Information).

The mesoderm not only gives rise to skeletal muscle but also develops into adipose and connective tissues.^[^ ^25^ ^]^ The development of mesoderm was enhanced in the MO embryos as represented by upregulated gene sets of “mesoderm morphogenesis” and “mesoderm development” (Figure 2D). However, genes related to “muscle tissue development”, “muscle organ development”, “muscle cell differentiation”, and “muscle adaption” were downregulated in MO offspring, suggesting that myogenic progress was impaired with MO (Figure 2A). Those down‐regulated myogenic genes included a myogenic differentiation determinator, Myog, and a list of structural proteins constituting mature myofibers such as Actc1, Myl2, Myl3, Mylpf, Tnnc1, and Tnni1 (Figure 2A and Table S2, Supporting Information). Meanwhile, gene sets involved in the “extracellular structure organization” and “cell‐to‐cell junctions” were up‐regulated in MO embryos (Figure 2D, Figure S2B and Table S4, Supporting Information), the dysregulation of which are responsible for collagen deposition in metabolic syndromes and cancer.^[^ ^26^, ^27^ ^]^ Therefore, our data suggests that MO reduced the myogenic process of mesodermal cells while enhanced their non‐myogenic progression.

In summary, our data suggest that MO induced metabolic dysfunctions in E9.5 embryos accompanied with oxidative stress, gene silencing, and chromatin remodeling, which are all possible outcomes of reduced oxygen transport and systematic hypoxia.

### 2.3 MO Enhances BMP Signaling within the DM

Embryonic myogenesis occurs within the DM under the control of regulatory signals from its surrounding tissues.^[^ ^19^, ^20^, ^21^ ^]^ MO‐induced disruptions to the local microenvironment might affect the biological process of embryonic myofiber formation which could contribute to impaired myogenic progress in offspring. Therefore, the underlying associations between MO‐induced changes and impaired muscle development within the DM were further explored. All cells of the DM cluster were then computationally segregated to evaluate MO‐induced changes. Consistent with systematic impairments to the total cell population of the embryo (Figure 2A,C), genes related to gas transport and cellular respiration were reduced while genes related to extracellular matrix organization and gene silencing increased in the DM (Figure S3A,B and Tables S5–S7, Supporting Information).

**Figure 3:**
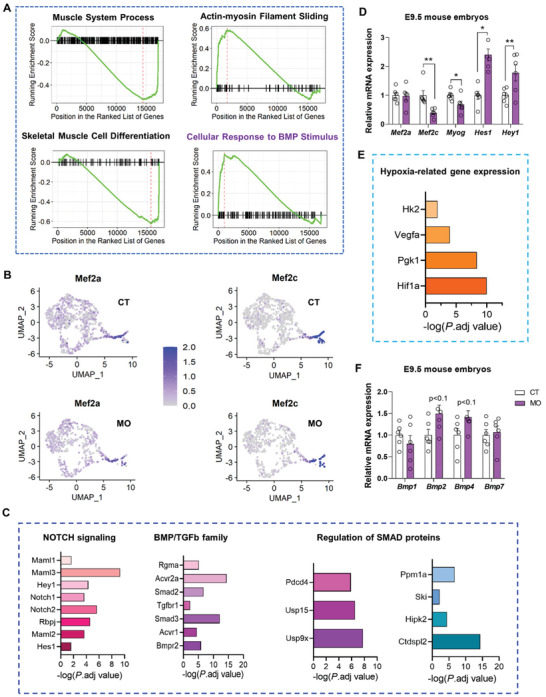
MO impairs myogenic processes within the DM. A) Representative terms of biological processes that are enriched by GSEA specific in the DM of the MO group. B) Feature plots showing the distribution of Mef2c and Mef2a in the DM. C) Representative genes with up‐regulated expression in the DM of the MO group that are involved in cellular response to BMP signals. D) Relative mRNA expression in E9.5 mouse embryos. E) Representative genes with up‐regulated expression in the DM of the MO group that are involved in hypoxic signaling and glycolysis. F) Relative mRNA expression in E9.5 mouse embryos. For (D,F), three embryos from each litter were pooled together for mRNA extraction and the litter was an experimental unit. *N* = 6. **p < 0.01, *p < 0.05 (two tailed *t*‐test), mean ± SEM. CT, the control group. MO, the maternal obese group. For (C) and (D), the *P*.adj (Benjamini–Hochberg adjusted *p* value, MAST test) < 0.05.

A coordinated down‐regulation of multiple gene sets related to the myogenesis was also observed in the MO group (**Figure** **3**A and Table S5, Supporting Information), including a 63% reduction of Mef2c and a 10% reduction of Mef2a (Figure 3B). MEF2C and MEF2A cooperate with MRFs to regulate myogenic differentiation.^[^ ^28^ ^]^ Within the DM, the distribution of Mef2c transcripts was predominantly restricted to a subset of cells while Mef2a was more widespread (Figure 3B). Moreover, a gene set related to “Cellular response to BMP stimulus” was enhanced in the DM (Figure 3A and Table S5, Supporting Information). Increased BMP signals have been reported to induce and enhance Notch signaling.^[^ ^29^, ^30^, ^31^, ^32^, ^33^ ^]^ Consistently, expression of Notch signaling related genes such as Notch1, Notch2, Rbpj, Maml1, Maml2, Maml3, Hey1, and Hes1 were increased in the DM of the MO group (Figure 3C), suggesting a synergistic relationship between Notch and BMP signaling in MO embryos. BMPs belong to the TGF*β* superfamily which inhibit myogenesis.^[^ ^34^, ^35^ ^]^ Gene expression involved in BMP/TGF*β* signaling, such as receptors (Tgfbr1, Acvr1, Acvr2a, Bmpr2) and effectors (Smad2 and Smad3), were increased in the MO group (Figure 3C). Meanwhile, genes that encode proteins with post‐translational regulatory roles on SMAD proteins (Usp15, Usp9x, Pdcd4, Ppm1a, Ski, Hipk2, and Ctdspl2) were also up‐regulated consistent with increased BMP signaling in the MO group. Reduced expression of Mef2c and Myog as well as enhanced expression of Hes1 and Hey1 by single‐cell transcriptome analysis were verified by RT‐qPCR of E9.5 embryo samples (Figure 3D). However, no difference was found for the expression of Mef2a (*p* > 0.05). In addition, MO also increased the expression of Hif1a, its target gene Vegfa, and glycolytic related genes Hk2 and Pgk1 (Figure 3E). As HIF1A enhances BMP signaling, MO‐induced hypoxic state within the DM may further aggravate the impairment of myogenesis.^[^ ^36^, ^37^ ^]^

Tissues surrounding the DM secrete BMPs, which play important roles in the specification and differentiation of cells within the DM.^[^ ^21^, ^38^, ^39^ ^]^ The expression of Bmp1 (*p* < 0.1) and Bmp7 in the somatopleure were increased in the MO group (Figure S3C, Supporting Information). The expression of Bmp4 and Bmp7 were also noticeably enhanced in the NOTO of the MO group though not statistically different (*p* > 0.05, Figure S3D, Supporting Information). Consistently, increased expression of Bmp2 (*p* < 0.1) and Bmp4 (*p* < 0.1) was also detected by RT‐qPCR analysis of E9.5 embryos (Figure 3F). These data suggest that increases of Bmp gene expression might contribute to the increased BMP signaling within the DM.

Overall, our data indicate a negative correlation between enhanced BMP signaling responses and impaired myogenic processes within the DM in response to MO.

### 2.4 Cellular Heterogeneity of the DM

**Figure 4:**
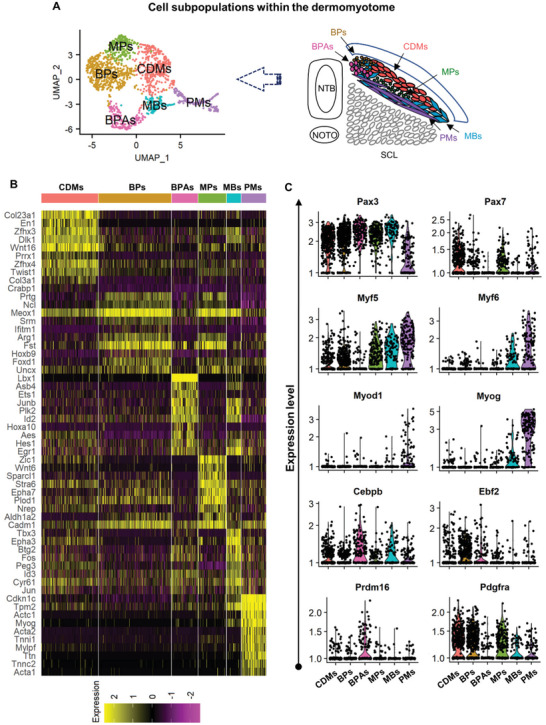
Single‐cell transcriptomic profiling of myogenic and brown adipogenic lineages identified in the DM. A) Through unsupervised clustering, cells in the DM were further sub‐divided into 6 populations and their distribution within the DM in vivo was grouped by marker gene expression. B) Heatmap showing the top 10 expressed genes within each cell population. C) VlnPlot showing the expression of manually selected canonical myogenic and brown adipogenic related genes across different cell populations. CDMs, central dermomyotomal cells; MPs, myogenic progenitors; MBs, myoblasts; PMs, primary myofibers; BPs, brown adipogenic progenitors; BPAs, brown preadipocytes; NTB, neural tube; NOTO, notochord; SCL, sclerotome.

The DM consists of not only cells involved in myogenic lineages, but also progenitor cells for the development of brown adipocytes. A better resolution of myogenic lineages within the DM was needed to elucidate the molecular mechanisms underlying the negative correlation between enhanced BMP signaling responses and impaired myogenic processes. To explore this further, we divided cells of the DM by unsupervised clustering and annotated 6 sub‐populations based on the expression of canonical marker genes (**Figure** **4**A and Table S8, Supporting Information). The cluster of central dermomyotomal cells (CDMs) was identified by the expression of Pax3, Pax7, Twist1, Twist2, Dlk1, and En1 (Figure 4B,C and Figure S4A, Supporting Information), which likely contained undifferentiated dermomyotomal cells and specified progenitors for the development of the dermis.^[^ ^40^ ^]^ The clusters of MPs were distinguished by the expression of Pax7 and brown adipogenic progenitors (BPs) by the expression of Ebf2 (Figure 4C). In brown preadipocytes (BPAs), Prdm16, Cebpb, and Ppargc1a expression was negatively correlated with the expression of Ebf2, suggesting a progressive commitment and differentiation of BP lineage. Cells in the cluster of myoblasts (MBs) expressed Pax3, Myf5, and Myf6 while the PMs expressed high levels of Myf6, Myog, Actc1, Myl1, Myh3, and Myh7 (Figure 4B,C and Figure S4B, Supporting Information).

The expression of well‐known regulatory genes on the myogenic specification including Meox1, Meox2, Six1, Six4, and Eya1 had similar distributions within the brown adipogenic and myogenic lineages while the expression of Eya2 was more specific to myogenic lineages (Figure S4C, Supporting Information).^[^ ^15^ ^]^ The expression of Pdgfra, which is a marker for mesenchymal stem cells and adipocyte progenitors, was highly expressed in cell populations of CDMs, BPs, and MPs, yet decreased in more differentiated populations (Figure 4C). The expression of Met and Lbx1 is important for the delamination of MPs from the central DM and for their migration to limb regions.^[^ ^41^, ^42^ ^]^ While the expression of Met was detected in the MBs, high expression of both Met and Lbx1 was detected in the BPAs, suggesting the delamination and migratory process of brown adipogenic lineages follows a similar mechanism with that of myogenic lineages (Figure S4D, Supporting Information).

In summary, our single‐cell transcriptomic profiling of the DM identified sub‐populations involved in both brown adipocyte and skeletal muscle lineages, revealing insights into lineage commitment and early differentiation of DM during embryonic development.

### 2.5 Reconstructing Embryonic Myogenic Process In Vivo

**Figure 5:**
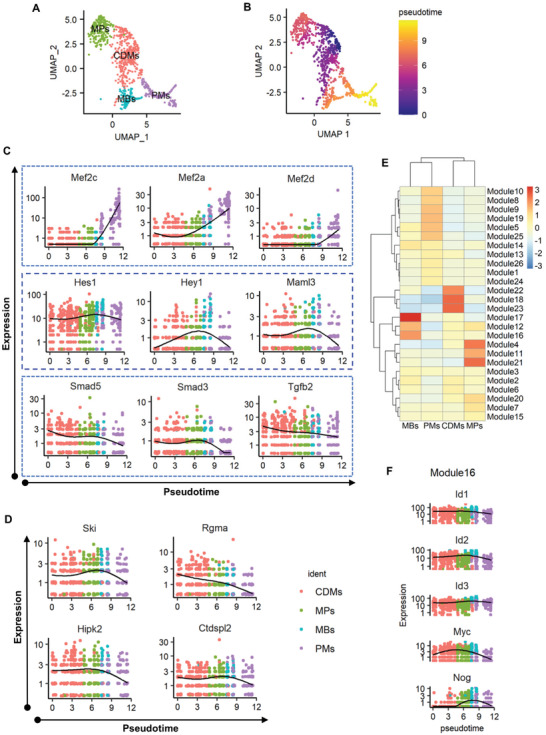
Re‐constructing the myogenic trajectory in vivo. A) Cells involved in myogenic lineages were selected; and B) used for trajectory construction by Monocle 3. C) Pseudotemporal changes of Mef2 family members and genes interacting with BMP signaling through the myogenic trajectory. D) Pseudotemporal changes of BMP signaling regulators through the pseudo myogenic path. E) Variable genes with changes as a function of pseudo time were grouped into 26 modules. F) Pseudotemporal changes of representative genes detected in Module 16. CDMs, central dermomyotomal cells; MPs, myogenic progenitors; MBs, myoblasts; PMs, primary myofibers.

To investigate regulatory mechanisms involved in the primary myogenic process in vivo, all cells of myogenic lineages within the DM were computationally isolated (**Figure** **5**A) for pseudo‐developmental trajectory analysis (Figure 5B) from undifferentiated CDMs to mature PMs. We first evaluated the dynamic changes of myogenic regulatory genes across the resulting pseudo‐developmental timeline (Figure S5A, Supporting Information). Consistent with their known functions during the myogenic differentiation process, the expression of Pax3 and Pax7 was gradually down‐regulated while Myf5, Myf6, Myod1, and Myog were up‐regulated across through pseudotime (Figure S5A, Supporting Information).

Next, we further analyzed pseudo‐temporal changes for genes of the Mef2 family to understand impairments within the DM caused by MO. Expression of Mef2a, Mef2c, and Mef2d all increased through myogenic differentiation (Figure 5C). Expression of Mef2b was not detected at this stage. Mef2c was the dominant isoform with restricted expression in more differentiated and fused PMs, while Mef2a was more universally distributed and expressed during the early stage of the myogenic process (Figures 5C and 3B). Therefore, our analyses suggest that Mef2c contributes primarily to myogenic differentiation and fusion at embryonic stages. Strikingly, Mef2c was 63% down‐regulated within the DM (Figure 3B), suggesting that Mef2c suppression might be a key factor leading to defects of embryonic muscle development of MO embryos.

Pseudo‐temporal changes of genes related to BMP signaling were also explored for their possible functions at a specific stage of myogenic process. High expression of Hes1, Hey1, and Maml3 were detected in MPs within CDMs and continuously down‐regulated during the commitment and differentiation of MBs to form myofibers (Figure 5C). Interestingly, the down‐regulation of Notch signaling effectors (Hes1, Hey1, and Maml3) coincided with the up‐regulation of Mef2c (Figure 5C), consistent with the roles of HES1 and HEY1 as transcriptional repressors. We also observed continuous decreases in the expression of Tgfb2 and its effector, Smad3, during the whole myogenic process (Figure 5C). Supportively, down‐regulation of TGF*β* signaling was recently identified as necessary for MB fusion within demomyotomal cells of the chicken.^[^ ^43^ ^]^ Similarly, the expression of BMP signaling effector, Smad5, decreased sharply during the commitment and differentiation of MBs along with gradual reduction of Smad4 and Bmpr2 (Figure 5C and Figure S5B, Supporting Information). Down‐regulation of BMP signaling and SMAD protein regulators such as Rgma, Hipk2, Ski, and Ctdspl2 were also found during the late stage of myogenic differentiation (Figure 5D). Taken together, a coordinated down‐regulation of BMP signaling was detected during the commitment and differentiation of myogenic cells in vivo.

To create a more complete overview of regulatory mechanisms across through pseudotime, gene expression was stratified into 26 modules (Figure 5E). Modular changes in gene expression within cell subtypes of the DM suggest stage‐specific functions during the myogenic process. Expression of Hes1 and Hey1 was grouped in module 16 along with other genes with lower expression in myofibers compared to MBs (Figure 5F and Table S9, Supporting Information). Among which, expression of Id1, Id2, and Id3, which are critical down‐stream transcriptional targets of BMPs, were all higher in MBs compared to myofibers (Figure 5F). The expression of Nog was activated to antagonize BMP signaling.^[^ ^44^ ^]^ Myogenic differentiation is more vulnerable to the inhibition of BMP signaling than the myogenic commitment of MP cells within the DM.^[^ ^21^ ^]^ These collective findings suggest that the decreased expression of Nog may potentiate the inhibitory functions of BMPs during myogenic differentiation. In addition, the expression of Myc, an inhibitor for myogenic differentiation, was also decreased.

Expression of Mef2c and multiple muscle structural genes such as Myl1, Actc1, and Acta2 continuously increased during the specification and differentiation of myogenic cells (Table S10, Supporting Information). Along with these genes, Cdkn1c, Eno3, Lgals1, Tpm1, and Tpm2 expression was consistently up‐regulated (Figure S5C, Supporting Information).

Taken together, our in silico reconstruction of the myogenic trajectory appears to accurately recapitulate the in vivo biological process. Of prominence, we found that the down‐regulation of transcriptional repressors, Hes1 and Hey1, coincided with the increase of Mef2c. Furthermore, MO increased expression of Hes1 and Hey1 in the DM while the expression of Mef2c was down‐regulated. This negative correlation suggests a potential link between Hes1/Hey1 and Mef2c that may explain MO‐induced myogenic retardation during embryonic development.

### 2.6 HES1/HEY1 Represses the Transcription of Mef2c during Myogenesis

**Figure 6:**
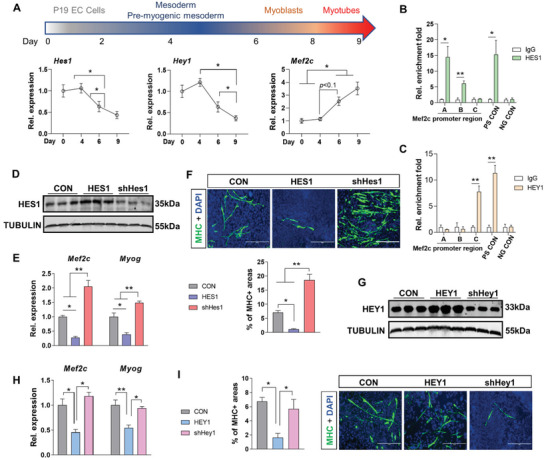
HES1 and HEY1 represses myogenic differentiation through the regulation of Mef2c. A) A timeline of myogenic differentiation in the P19 EC cells. Relative mRNA expression of Hes1, Hey1, and Mef2c was shown across the timeline. ChIP‐qPCR analysis of B) HES1‐ or C) HEY1‐bound Mef2c promoter regions, and their positive (PS) and negative (NG) controls (CON), respectively. Relative enrichment folds were normalized to the IgG group. D) Immunoblotting of HES1 in transfected P19 cells at day 6 of myogenic induction. CON, control; HES1, overexpression of HES1 open reading frame; shHes1, shRNA of Hes1. E) Relative mRNA expression of Mef2c and Myog at day 6 of myogenic induction in transfected P19 cells (D). F) Immunofluorescence of MHC in transfected P19 cells after 9 days of differentiation (D). The percentage of MHC+ areas were measured. Bar, 1:200 µm. G) Immunoblotting of HEY1 in transfected P19 cells at day 6 of myogenic induction. CON, control; HEY1, overexpression of HEY1 open reading frame; shHey1, shRNA of Hey1. H) Relative mRNA expression of Mef2c and Myog at day 6 of myogenic induction in transfected P19 cells (G). I) Immunofluorescence of MHC in transfected P19 cells after 9 days of differentiation (G). The percentage of MHC+ areas were measured. Bar, 1:200 µm. For statistical analysis, the one‐way AVOVA followed by Bonferroni correction was used for (A), (E), (F), (H), and (I) while the two tailed *t*‐test was used for (B) and (C). **p < 0.01, *p < 0.05, mean ± SEM; *N*  =  3.

An embryonic carcinoma (EC) cell line (P19 cells) is commonly used to model embryonic myogenic progress in vitro (**Figure** **6**A).^[^ ^45^ ^]^ Therefore, using the P19 cells, we further explored the mechanistic framework underlying the inverse relationship between Hes1/Hey1 and Mef2c during an induced myogenic process. The expression of Hes1 and Hey1 were up‐regulated during the first 4 days of differentiation in vitro before significantly declining (Figure 6A). In contrast, the expression of Mef2c gradually increased during myogenic differentiation. Collectively, in vitro differentiation studies accurately recapitulated the inverse relationship between Hes1/Hey1 and Mef2c observed in vivo within mouse embryos.

To determine if HES1 or HEY1 bind to regulatory regions within the Mef2c promoter, P19 cells were collected at day 3 (pre‐myogenic mesoderm, Figure 6A) of in vitro differentiation for ChIP‐PCR analysis. Outcomes from ChIP pull‐down identified two binding sites for HES1 and one binding site for HEY1 within the Mef2c promoter region, suggesting that HES1/HEY1 directly control Mef2c transcription (Figure 6B,C).

Next, we explored the mediatory function of HES1 and HEY1 on the transcription of Mef2c and myogenic differentiation using gain and loss of function approaches (Figure 6D and Figure S6A, Supporting Information). At day 6 (MBs, Figure 6A) of in vitro differentiation, over‐expression of HES1 decreased the expression of Mef2c and its direct target, Myog, while knock‐down of HES1 up‐regulated their expression (Figure 6E). Consistently, over‐expression of HES1 blocked MHC+ myotube formation at day 9 (myotubes, Figure 6A) in vitro while knock‐down of HES1 enhanced myotube formation (Figure 6F). Similarly, over‐expression of HEY1 (Figure 6G and Figure S6B, Supporting Information) also suppressed the expression of Mef2c and Myog (Figure 6H) with a concomitant block in MHC+ myotube formation (Figure 6I). However, knock‐down of HEY1 did not increase Mef2c and Myog expression or MHC+ myotube formation, which may indicate potential functional redundancy of HEY1 with other HEY family members in myogenesis consistent with previous reports.^[^ ^46^ ^]^

In summary, our data showed that enhanced expression of HES1 and HEY1 repressed the myogenic differentiation through transcriptionally repressing the expression of Mef2c. Knockdown of HES1 but not HEY1 promoted expression of Mef2c and myogenesis as functional redundancies may exist for HEY1. MO‐promoted expression of Hes1 and Hey1 explains the decreased expression of Mef2c and impaired myogenic process in E9.5 embryos of mice.

### 2.7 Increased Hypoxia Blocked Myogenesis through Upregulating HES1

**Figure 7:**
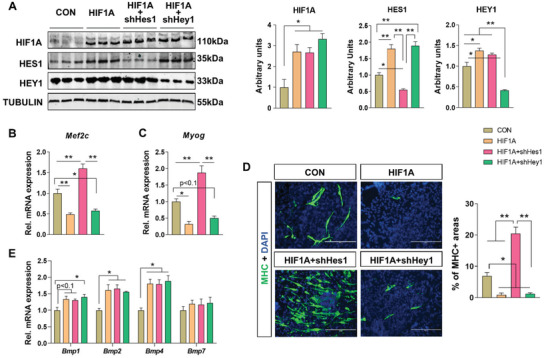
HIF1A represses myogenic differentiation through its regulation on HES1 and HEY1. A) Immunoblotting and arbitrary units of HIF1A, HES1, and HEY1 in transfected P19 cells at day 6 of myogenic induction. B,C) Relative mRNA expression of Mef2c and Myog at day 6 of myogenic induction in transfected P19 cells. D) Immunofluorescence of MHC in transfected P19 cells after 9 days of differentiation. The percentage of MHC+ areas were measured. Bar, 1:200 µm. E) Relative mRNA expression of BMP ligands at day 6 of myogenic induction in transfected P19 cells. CON, control; HIF1A, overexpression of HIF1A; HIF1A + shHes1, overexpression of HIF1A with knocked‐down expression of HES1; HIF1A + shHey1, overexpression of HIF1A with knocked‐down expression of HEY1. **p < 0.01, *p < 0.05 (one‐way ANOVA followed by Bonferroni correction), mean ± SEM; *N*  =  3.

Because hypoxia is known to suppress myogenesis and MO enhances hypoxia, we further explored the associations between HIF1A, HES1/HEY1, and myogenic differentiation.^[^ ^47^ ^]^ With over‐expression of a normoxia‐stable HIF1A in the P19 cells (**Figure** **7**A), Mef2c expression was blocked along with suppressed expression of Myog (Figure 7B,C) and almost complete absence of MHC+ myotubes (Figure 7D). Over‐expression of HIF1A also elevated the protein levels of HES1 and HEY1 at day 6 of the myogenic process (Figure 7A). Interestingly, the expression of Mef2c and Myog at day 6 and MHC+ myotube formation at day 9 were recovered and enhanced after knocking‐down of HES1 but not the HEY1 (Figure 7B–D), suggesting that HES1 serves as an important down‐stream effector of HIF1A on myogenic suppression. Meanwhile, the expression of Bmp1 (*p* < 0.1), Bmp2, and Bmp4 was also up‐regulated with HIF1A over‐expression compared to CON (Figure 7E). Therefore, our data suggest that HIF1A enhances BMP signaling and the expression of HES1/HEY1, which suppresses the expression of Mef2c and myogenic differentiation.

### 2.8 MO‐Induced Stage‐Specific Impairments to Myogenic Subpopulations and its Potential Links with Skeletal Muscle Dysfunctions Later in Life

While PMs serve as the scaffold for the fetal myofiber formation, MPs and MBs formed within the DM also contribute to skeletal muscle development at fetal and postnatal stages.^[^ ^48^ ^]^ MO‐altered changes in those embryonic myogenic lineages may have unique associations with the development of skeletal muscle disorders in offspring. Therefore, we further explored the stage specific disruptions of MO to myogenic subpopulations to better understand their potential links with skeletal muscle dysfunctions in later life.

**Figure 8:**
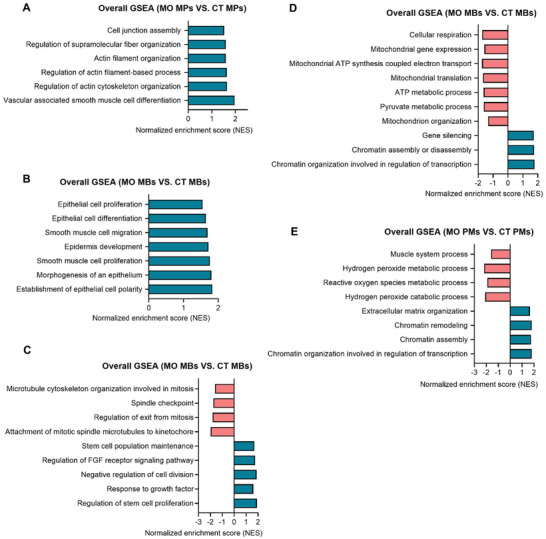
Stage‐specific disruptions to myogenic lineages due to MO. A) Representative terms of biological processes that were enriched by GSEA in MPs within the DM of the MO group. B–D) Representative terms of biological processes that were enriched by GSEA in MBs within the DM of the MO group. E) Representative terms of biological processes that were enriched by GSEA in PMs within the DM of the MO group. DM, dermomyotome; MPs, myogenic progenitors; MBs, myoblasts; PMs, primary myofibers; CT, the control group; MO, the maternal obese group; GSEA, gene set enrichment analysis.

A gene set of “Vascular‐associated smooth muscle cell differentiation” was found to be up‐regulated in MPs (Figure 4A) of the MO group (**Figure** **8**A and Table S11, Supporting Information). Enhanced smooth muscle related gene expression represses skeletal myogenic progress as smooth and skeletal muscle cells mutually transdifferentiate in early embryos.^[^ ^49^ ^]^ Smooth muscle cells are closely related to the extracellular matrix organization.^[^ ^50^ ^]^ Consistently, multiple gene sets related to extracellular matrix organization were enhanced in the MPs of the MO group including gene sets of “Regulation of actin cytoskeleton organization”, “Regulation of actin filament‐based process”, and “Regulation of supramolecular fiber organization”. Up‐regulated gene sets of “Smooth muscle cell migration” and “Smooth muscle cell proliferation” were also identified in the MBs of the MO group (Figure 8B and Table S12, Supporting Information) along with an upregulated gene set of “extracellular matrix organization” in the PMs of the MO offspring (Figure 8E and Table S13, Supporting Information). Therefore, these data suggest that MO reduces myogenic potential of early progenitors and promotes non‐myogenic differentiation.

Gene sets related to epithelial cell development were identified within the MBs (Figure 4A) of MO embryos, suggesting their impaired transition to mesenchymal cells which is a necessary process for embryonic myogenic differentiation (Figure 8B).^[^ ^51^ ^]^ In addition, gene sets related to the mitotic process were down‐regulated in the MO MBs (Figure 8C). Moreover, up‐regulated gene sets of “Stem cell population maintenance”, “Regulation of stem cell proliferation”, and “Regulation of FGF receptor signaling pathway” in the MO MBs suggest that MO maintained MBs in a less differentiated state (Figure 8C). Embryonic MBs not only differentiate into PMs, but also proliferate and contribute directly to fetal myotube formation. Downregulated gene sets related to mitochondria functions such as “Cellular respiration” and “Mitochondrial ATP synthesis coupled electron transport” were observed in MBs of the MO group (Figure 8D). Because a glycolytic to oxidative transition is needed for myogenic differentiation, its suppression in the MO embryos should attenuate myogenic differentiation.^[^ ^52^ ^]^ Consistently, a gene set of “muscle system progress”, which include gene expression of Mef2c, Myog, Myod1, and muscle structural genes, was downregulated in the PMs (Figure 4A) of the MO group (Figure 8E). In addition, the muscle fiber shift from oxidative to glycolytic phenotypes due to MO might result in long‐term detrimental effects to the metabolic function of offspring skeletal muscle.^[^ ^53^ ^]^

In summary, our study suggest that MO reduces skeletal muscle formation by enhancing BMP signaling, attenuating embryonic myogenic differentiation while promoting a non‐myogenic potential of MPs. The impairment in embryonic myogenesis and attenuation of glycolytic and oxidative metabolic transition provide a mechanistic explanation for the subsequent fetal muscle development and the long‐term impairment in offspring muscle function due to MO.

## 3 Discussion

In humans, women with pre‐pregnancy obesity are more likely to gain excessive body weight during gestation, which increases the risks of developing pregnancy complications such as gestational diabetes and thus affects the health of both mothers and their fetuses.^[^ ^54^, ^55^ ^]^ Both maternal pre‐pregnancy obesity and excessive gestational body weight gain are closely related with increased adiposity and its associated metabolic dysfunctions in offspring.^[^ ^56^, ^57^, ^58^ ^]^ Interestingly, clinical management to avoid excessive body weight gain during gestation in obese women alleviates those adverse effects in children.^[^ ^54^, ^55^ ^]^ However, prevention of obesity before pregnancy is still recommended as detrimental effects of MO starts early during pregnancy and even in oocytes.^[^ ^55^, ^59^, ^60^ ^]^ Although molecular mechanisms linking MO to adverse changes in offspring remain to be fully defined, changes in intra‐uterine environment is one of the leading causes for health disruption in offspring.^[^ ^4^ ^]^ In effectively mimicking obesity in pregnant women, a diet‐induced MO model which includes both pre‐gestational and gestational obesity was used in the current study.

MO during pregnancy is associated with disrupted oxygen and nutrient delivery from the mother to the fetus, impairing fetal development.^[^ ^61^ ^]^ In both human and animal studies, MO reduces vascularity and constrains blood flow in the placenta.^[^ ^62^, ^63^ ^]^ Consistently, we found that MO decreased the expression of oxygen transporters, the hemoglobin‐related genes, and increased the expression of Hif1*α* during embryonic myogenesis, suggesting a general hypoxic state with MO. Chronic hypoxia stabilizes HIF1A, which up‐regulates the expression of glycolytic genes while suppressing genes involved in oxidative metabolism.^[^ ^64^ ^]^ Hypoxia‐induced mitochondrial dysfunction further aggravates oxidative stress and inflammation.^[^ ^65^ ^]^ In this study, we found that MO increased gene expression involved in chromatin re‐organization and gene silencing, consistent with hypoxia‐induced changes.^[^ ^66^ ^]^ These epigenetic modifications due to MO potentially predispose offspring to long‐term metabolic disruptions.^[^ ^67^ ^]^

Accompanied with impaired metabolic transition in the embryos of MO group, skeletal muscle development was impaired. Elevated BMP signaling responses were found specifically within the DM of MO embryos. The BMP signals are known for their important regulatory functions in bone formation, and osteogenic cells are originally derived from progenitors within the other compartment of a mature somite, the SCL.^[^ ^14^, ^68^ ^]^ Disrupted BMP signaling affects the proliferation and differentiation of osteoblasts and chondrocytes which underlies the development of multiple human skeletal disorders.^[^ ^68^ ^]^ In addition, enhanced BMP signaling also encourages adipogenic differentiation of progenitor cells,^[^ ^69^ ^]^ consistent with up‐regulation of non‐myogenic differentiation observed in MO embryos. Considering that BMP signaling attenuates during the differentiation of embryonic myofibers in a pseudo‐myogenic process observed in this study, the BMP elevation in the DM of MO embryos might block myogenesis. Consistent with this notion, a low BMP4 level in the lateral plate mesoderm increases the expression of Pax3, an early marker of myogenic commitment within the DM.^[^ ^21^ ^]^ Increased levels of BMPs suppress the expression of Pax3 and regulators of myogenic differentiation within the DM.^[^ ^21^ ^]^ Hypoxic condition and expression of Hif1a amplify BMP signaling by increasing BMP ligand expression and down‐stream signaling.^[^ ^36^, ^37^ ^]^ In agreement, we found that over‐expression of HIF1A increased BMP ligand expression in P19 cells.

BMP signaling is known to synergize with the Notch signaling.^[^ ^29^, ^30^, ^31^, ^32^, ^33^ ^]^ Activation of BMP signaling in C2C12 cells promotes the expression of Hes1 and Hey1, two key down‐stream mediators of Notch signaling.^[^ ^29^ ^]^ Furthermore, binding sites for SMAD proteins, the downstream effectors of BMP signaling, are located within the Hey1 promoter.^[^ ^29^, ^33^, ^70^ ^]^ HES1 functions as a transcription repressor which forms homodimers or heterodimers with HEY1 or HEYL.^[^ ^46^ ^]^ Over‐expression of HES1, HEY1, and HEYL in adult myogenic stem cells (satellite cells) suppresses myogenic differentiation with redundancies between HEY1 and HEYL for binding with HES1.^[^ ^46^ ^]^ Similarly, our studies in P19 cells demonstrate the inhibitory roles of HES1 and HEY1 in myogenic differentiation, which were achieved through suppression of Mef2c transcription. MEF2 proteins bind and potentiate the myogenic function of MRFs and myogenesis.^[^ ^28^ ^]^ Using our pseudo‐myogenic model, the expression of Mef2c was correlated with the up‐regulation of Myf6 and Myog, suggesting its essential roles for the embryonic myogenesis within the DM. Furthermore, while over‐expression of HIF1A increased the protein levels of both HES1 and HEY1, HES1 but not HEY1 was indispensable for HIF1A‐induced myogenic inhibition as redundancy exists for HEY1. In addition to its induction of BMP ligands, HIF1A also enhances the expression of Hes1.^[^ ^71^ ^]^ Therefore, our data suggest that MO‐induced hypoxia maintains a high level of BMP signaling within the DM, which suppresses the expression of Mef2c and myogenic differentiation through up‐regulation of Hes1and Hey1.

In addition, brown adipocytes are also derived from MYF5+ progenitors within the DM.^[^ ^72^, ^73^ ^]^ For the first time, to our knowledge, the BPs and BPAs within the DM were profiled, which provided valuable information about the biological process of brown adipocyte development within the DM.

MO induced stage‐specific impairments to myogenic lineages. Enhanced smooth muscle cell related biological processes within MPs suggest their potential transition to non‐myogenic lineages in response to MO.^[^ ^49^ ^]^ Extracellular matrix is enhanced by the smooth muscle cells, which is associated with enhanced fibrosis formation within the skeletal muscle and the development of muscle diseases and disorders later in life.^[^ ^11^, ^50^ ^]^ Consistent with our finding of impaired skeletal muscle cell differentiation resulted from MO‐reduced Mef2c expression, MO maintained an undifferentiated state of embryonic MBs through reducing epithelial to mesenchymal transition and enhancing FGF responses. Collectively, those changes resulted in downregulated myogenic system progress in embryonic myofibers of the MO group. As embryonic myofibers form template for fetal and postnatal myogenesis, those early changes might contribute to reduced numbers and strength of myofibers in offspring. Moreover, reduced mitochondria function was also detected in the embryonic MBs. The metabolic phenotype of myofibers was determined by the metabolic state of MBs before the myotube formation.^[^ ^74^ ^]^ As embryonic MBs develop into both embryonic myofibers and fetal myofibers, reduced mitochondria function in these MBs of the MO group might lead to the shift of myofibers from oxidative to glycolytic phenotype. As MEF2C promotes an oxidative muscle fiber type,^[^ ^75^ ^]^ reduced expression of Mef2c might also contribute to this metabolic phenotype transition of myofibers. Gene expression changes caused by gene silencing and chromatin remodeling in MBs and myofibers further increase risks of muscle diseases and disorders in offspring.

## 4 Conclusion

In conclusion, our single‐cell transcriptomic analysis of E9.5 embryos revealed that MO‐induced hypoxia contributes to multiple impairments to the embryos especially the development of skeletal muscle. MO increases cellular responses to BMP stimuli, which suppresses the expression of Mef2c through the up‐regulation of Hes1 and Hey1, impairing myogenic differentiation within the DM. Stage specific disruptions of MO to myogenic lineages elucidate the progressive impairments to the skeletal muscle development, which exert long‐term effects to the function and metabolic health of the skeletal muscle. In addition, our study also provides novel information of embryonic organogenesis and myogenic related biological processes, and deepen mechanistic understanding of how MO impairs embryonic myogenesis, which is clinically important considering the commonness of MO.

## 5 Experimental Section

### Experimental Design

Wild type C57BL/6J mice were originally purchased from the Jackson Laboratory (Bar Harbor Maine). All animal studies were conducted in AAALAC‐approved facilities under the guideline of the Institutional Animal Use and Care Committee at Washington State University (Permit No. 6704). To induce MO, female mice at 8 weeks of age were randomly separated into two groups and fed with either HFD (45% energy from fat, D12451; Research Diets, New Brunswick, NJ) or CD (10% energy from fat, D12450, Research Diets) for 10 weeks. Adult C57BL/6J males (3‐month‐old) maintained on a regular chow were used to mate with those females. Vaginal plug checking was conducted in the early morning and the day a vaginal plug observed was designated as E0.5. During pregnancy, female mice were continued on their respective diets. Mouse embryos were dissected and collected on E9.5. Single cell suspensions from collected embryo samples were applied to scRNA‐seq pipelines. In addition, embryonic skeletal myogenesis was modeled by induced myogenesis of P19 EC cells.

### Embryo Collection and Single‐Cell RNA‐Sequencing

To minimize developmental differences between embryos, the developmental stage was confirmed according to the morphological criteria of Downs and Davies, and Theiler Stages (Stage 15), and only embryos with 23–26 somite pairs observed under a dissecting microscope (Leica DFC450 C) were selected.^[^ ^76^, ^77^ ^]^ After removing the portion of head and first 7 somite pairs, the portion starting from the 8th somite pair till the tail end was collected.^[^ ^76^ ^]^ No internal organs or tissues was removed. For each sample, a total of 15 embryos from three litters of MO or lean (CT) mice, respectively, were pooled together for the preparation of cell suspension. One sample for the CT or MO was collected on the same day and another sample collected on a second day. Similar sampling procedures were used in other studies.^[^ ^78^ ^]^

After mincing, embryo samples were incubated with TryLE Express dissociation reagent (Life Technology) at 37 °C for 15 min, swirled every two minutes, and the reaction was quenched with heat inactivated serum. After washing, the dissociated single‐cell suspensions were re‐suspended in PBS with 0.04% BSA and filtered through a 40 µm cell strainer. After checking the viability and counting, single‐cell suspensions of each group were loaded onto the Chromium Controller and cDNA libraries were constructed using the Chromium Single Cell 3’ Library & Gel Bead Kit v3 following the manufacturer's protocol (10X Genomics, Inc.). All samples were multiplexed together and sequenced across one single lane of an Illumina NovaSeq 6000 S4 (Genewiz, South Plainfield, NJ).

### Data Analysis

Raw sequencing data were pre‐processed with Cell Ranger (v3.0.2, 10X Genomics) mapping to the mouse mm10 transcriptome. A total of 28 119 cells from all four libraries were integrated in R using the Seurat package (v3.2.1).^[^ ^79^ ^]^ For filtering low‐quality cell transcriptomes and doublets, cells with greater than 200 000 UMIs and with less than 500 genes per cell were excluded. Cells with mitochondrial gene expression greater than 20% were removed. After filtering, 25 021 cells were retained for downstream analysis. The resulting dataset was then normalized with a global‐scaling normalization method (“LogNormalize”) with default parameters. For dimensional reduction, the normalized dataset was further scaled using the “ScaleData” function. Variable genes identified using the function of “FindVariableGenes” were used to perform principal component analyses. The top 94 dimensions were selected for clustering (“resolution” set to 0.5) and uniform manifold approximation and projection (UMAP) graphing. Specific gene markers for each cluster were identified using the “FindAllMarkers” function. The SimplifyStats package (v1.0.1) was used to calculate cluster and treatment distribution. Differential gene expression between treatments was analyzed using the MAST test in “FindMarkers” function with Bonferroni adjusted *p* value < 0.05 showing significant differences. Modified multivariate Pearson's RV correlations were calculated for each set of treatment replicates using the MatrixCorrelation package (v0.9.2).^[^ ^80^ ^]^ The following correlations showed consistent sampling between replicate libraries: HFD1 and HFD2 = 0.81, CT1 and CT2 = 0.73, CT and HFD = 0.97. The function of “subset” was used to computationally select a specific cluster of cells. Gene Ontology (GO) and GSEA were performed with the R package, clusterProfiler v3.18.0, using all detected genes from the entire scRNA‐seq library as background.^[^ ^81^ ^]^ Terms were enriched with the nominal *p* value < 0.05 and false discovery rate (*q* value) < 0.05.

### Single‐Cell Trajectory Analysis

To investigate molecular regulations over the biological process of embryonic myogenesis, single‐cell trajectory analysis was performed by pseudo‐ordering the myogenic subpopulations according to their developmental timeline. The data matrix of Seurat objects was imported to Monocle3 for trajectory inference and pseudotime calculation.^[^ ^82^ ^]^ After making a new cell_data_set object, the overall trajectory of gene expression changes was constructed by the “learn_graph” function and cells along with this trajectory were plotted after ordering cells (“order_cells”). Gene sets with consistent variations across the pseudotime were grouped into modules using Louvain community analyses (“find_gene_modules”).

### Fasting Glucose and Insulin Test, and Glucose Tolerance Test

For fasting glucose and insulin tests, blood was collected from the tail vein of mice after 5 h of fasting. The concentration of glucose was measured by a glucometer (Bayer Contour, Tarrytown, NY, USA). The concentration of insulin was determined by a Mouse Ultrasensitive insulin ELISA Kit (ALPCO Diagnostics, Salem, NH, USA). HOMA‐IR was calculated using the following formula: HOMA‐IR = fasting insulin (mU L^−1^) × fasting blood glucose (mmol L^−1^)/22.5.^[^ ^83^ ^]^ In addition, a glucose tolerance test was performed after overnight fasting. After injecting the mice with D‐glucose (1 g/kg body weight, i.p.), blood was collected from the tail vein at 0, 15, 30, 60, 90, 120 min post‐injection for the measurement of glucose levels.

### Cell Line, Plasmid Transfection, and Assays

P19 EC cells were cultured and induced to myogenic differentiation according to previous reports.^[^ ^45^ ^]^ Briefly, cells were cultured in a growth media with 5% fetal bovine serum and 5% bovine calf serum in a minimal essential medium with *α* modification. Cells were aggregated in the presence of 0.8% DMSO with growth media in non‐coating petri dishes for 4 days before plating on cell culture dishes with growth media only. The first day of inducing 3D spheroid formation was considered as Day 0. Cells were collected at different time points after inducing myogenic differentiation and then used for RT‐qPCR and immunoblotting analyses.

For RNA interference, plasmid shHes1 (TRCN0000321465), shHey1 (TRCN0000374147), and control vector 1 (CV1, SHC201) were obtained from Sigma‐Aldrich (St. Louis, Missouri). For over‐expression analyses, plasmid HES1 open‐reading frame (HES1/ORF, Ex‐Mm03008‐M35), HEY1 ORF (HEY1/ORF, Ex‐Mm24690‐M35), and control vector 2 (CV2, Ex‐EGFP‐M35) were obtained from GeneCopoeia (Rockville, MD). Plasmid HIF1A (HIF1A, catalog no. 44 028) and control vector 3 (CV3, catalog no. 13 031) were obtained from Addgene (Cambridge, MA). To compare myogenic process regulated by HES1, P19 cells were co‐transfected with plasmids of CV1 and CV2 as the control group (CON), plasmids of CV1 and HES1/ORF as the HES1 overexpression group (HES1), and plasmids of shHes1 and CV2 as the HES1 knockdown group (shHes1). Similarly, to compare myogenic process regulated by HEY1, P19 cells were co‐transfected with plasmids of CV1 and CV2 as the control group (CON), plasmids of CV1 and HEY1/ORF as the HEY1 overexpression group (Hey1), and plasmids of shHey1 and CV2 as the HEY1 knockdown group (shHey1). To explore the mediatory roles of HES1 and HEY1 in myogenesis impaired by HIF1A over‐expression, P19 cells were co‐transfected with plasmids of CV1 and CV3 as the control group (CON), plasmids of CV1 and HIF1A as the HIF1A overexpression group (HIF1A), plasmids of HIF1A and shHes1 as the HES1 knockdown group (HIF1A+shHes1), and plasmids of HIF1A and shHey1 as the HEY1 knockdown group (HIF1A+shHey1). Transfection was performed using Lipofectamine 3000 transfection reagent (Invitrogen). Following infection, cells were treated with puromycin and neomycin for 2 d.

### qPCR Analyses

Total RNA was isolated using TRIzol reagent (Sigma #T9424). The cDNA templates were obtained from 500 ng of purified RNA using iScriptTM cDNA Synthesis kit (Bio‐Rad # 1 708 891). A SYBR Green RT‐PCR kit (Bio‐Rad # 1 725 274) was used for qPCR using a CFX RT‐PCR detection system (Bio‐Rad).^[^ ^28^ ^]^ 18S was used as the reference gene to normalize mRNA expression levels. Data were analyzed using 2‐ΔΔCt method. The primers used are listed in Table S14, Supporting Information.

### Immunoblotting Analysis

As previously described, immunoblotting analyses were performed using the Odyssey Infrared Image System (LICOR Biosciences, Lincoln, NE, USA). Primary antibodies included anti‐TUBULIN (CST, #2146), anti‐HES1 (Santa Cruz, #166 410), anti‐HEY1 (Proteintech, #19929‐1‐AP), and anti‐HIF1A (ThermoFisher, PA1‐16601) antibodies. The secondary antibodies, IRDye 800CW goat anti‐rabbit (# 926 32211) and IRDye 680RD goat anti‐mouse (# 926 68070), were purchased from LI‐COR Biosciences (Lincoln, NE, USA). Quantification was normalized according to the expression of *β*‐TUBULIN.

### Immunofluorescence Staining

Cells were fixed in cold methanol for 15 min at −20 °C, blocked with 10% serum for 1 h, and incubated with the primary antibody against myosin heavy chain (MHC, MF20, DSHB, AB_2 147 781) overnight at 4 °C. Cells were then stained with the corresponding secondary antibody for 1 h at room temperature. Nuclei were stained with DAPI in mounting medium (Vector Laboratories, Burlingame, CA). Images of the cells were taken using a fluorescence microscope (EVOS FL, Life Technologies).

### ChIP–qPCR Assays

At day 3 of differentiation, P19 cells were collected for ChIP‐qPCR assays as previously described with minor modifications.^[^ ^84^ ^]^ Briefly, trypsinized cells were fixed with 1% formaldehyde and incubated for 10 min with agitation. Cross‐linking was terminated by adding 125 mm glycine. Cell samples were then lysed in a cold lysis buffer (1% SDS, 10 mm Tris‐HCl, pH 8.0, 10 mm EDTA) supplemented with a protein inhibitor cocktail (Sigma, P2714). Then the samples were sonicated to produce 200–400 kb segments. A quantity of 60 µg sheared chromatin samples was incubated with antibodies against HES1, HEY1, or IgG non‐specific antibodies. The immune complexes were captured using ChIP‐grade Pierce magnetic protein A/G (Thermo Scientific, Waltham, MA). After washing, DNA was purified with Phenol: Chloroform: Isoamyl alcohol (25:24:1) (ACROS, A0417977). Obtained DNA was used for Real‐time qPCR. The primers are listed in Table S15, Supporting Information. To confirm specific binding, the enrichment of the same chromatin locus was compared between samples immune‐precipitated with a specific antibody and a non‐specific IgG.^[^ ^85^, ^86^ ^]^

### Statistical Analysis

For single‐cell transcriptomic sequencing, in each treatment, a total of 30 embryos from 6 litters were used for sample preparation. Pre‐processing of raw sequencing data including transformation, normalization, and quality control were described in the above “Data Analysis” section. For the analysis of differential gene expression, the MAST test was performed using the R package Seurat (v3.2.1). For RT‐qPCR of mouse embryos, three embryos from each litter were pooled for RNA extraction and 6 litters were used for each group. For in vitro cell experiments, statistical significance was detected by a two tailed *t*‐test, or a one‐way ANOVA followed by Bonferroni correction. Three independent studies were conducted for cell culture studies (*N* = 3). Data were analyzed with GraphPad Prism (version 7) and presented as means ± SEM. Statistical differences were indicated as *p < 0.05 and **p < 0.01.

## Conflict of Interest

The authors declare no conflict of interest.

## Supporting information

Supporting Information

## Data Availability

The data that support the findings of this study are openly available in GEO database at NCBI, reference number GSE173994.
