# Supplementary material for: Obesity Impairs Embryonic Myogenesis by Enhancing BMP Signaling within the Dermomyotome
Source: Adv Sci (Weinh). 2021 Oct 14;8(22):2102157. doi: 10.1002/advs.202102157 (PMC8596142; doi:10.1002/advs.202102157)
Supplement: Supplementary file 1 — Supporting Information [file ADVS-8-2102157-s001.pdf]

ADVANCED SCIENCE
Open Access

Supporting Information

for *Adv. Sci.*, DOI: 10.1002/advs.202102157

Obesity Impairs Embryonic Myogenesis by Enhancing BMP Signaling within the Dermomyotome

*Liang Zhao, Nathan C. Law, Noe A. Gomez, Junseok Son, Yao Gao, Xiangdong Liu, Jeanene M. de Avila, Mei-Jun Zhu, and Min Du**

Supporting Information

Title: Obesity impairs embryonic myogenesis by enhancing BMP signaling within the dermomyotome

*Liang Zhao, Nathan C. Law, Noe A. Gomez, Junseok Son, Yao Gao, Xiangdong Liu, Jeanene M. de Avila, Mei-Jun Zhu, and Min Du**

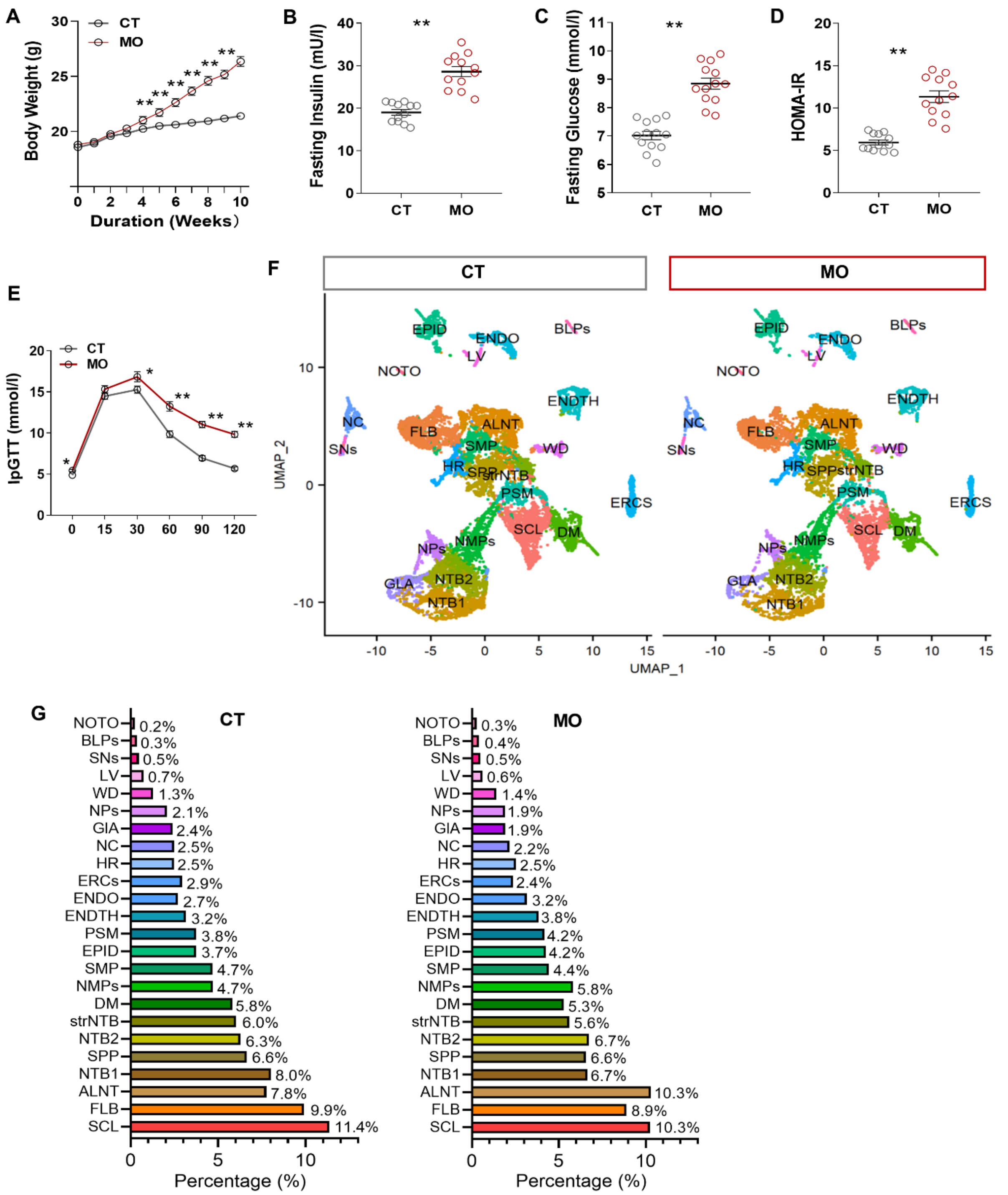

Figure S1. Single-cell transcriptomic analysis of E9.5 embryos affected by maternal obesity.

(A) Dynamic body weight changes of female mice in control (CT) and maternal obese (MO) groups.

(B-D) Fasting blood glucose, insulin, and calculated HOMA-IR in female mice before mating.

(E) Glucose tolerance test (GTT) in female mice before mating, after overnight fasting.

(F) UMAP visualization of clusters identified in the CT and MO samples, respectively.

(G) Proportions of each cluster in E9.5 embryos of the CT and MO samples, respectively. SCL, sclerotome; FLB, forelimb bud; ALNT, allantois; NTB1, neural tubes 1; SPP, splanchnopleure; NTB2, neural tubes 2; strNTB, stressed neural tubes; DM, dermomyotome;

NMPs, neuromesodermal progenitors; SMP, somatopleure; EPID, epidermis; PSM, pre-somitic mesoderm; ENDTH, endothelium; ENDO, endoderm; ERCS, erythroid cells; HR, heart; NC, neural crest; GLA, ganglia; NPs, Neural Crest; MSN, mesonephroi; LV, liver bud; SNs, sensory neurons; BLPs, blood progenitors; NOTO, notochord.
For A-E, data was represented as means ± SEM. $N = 12$. *$P < 0.05$; **$P < 0.01$ (two tailed t-test).

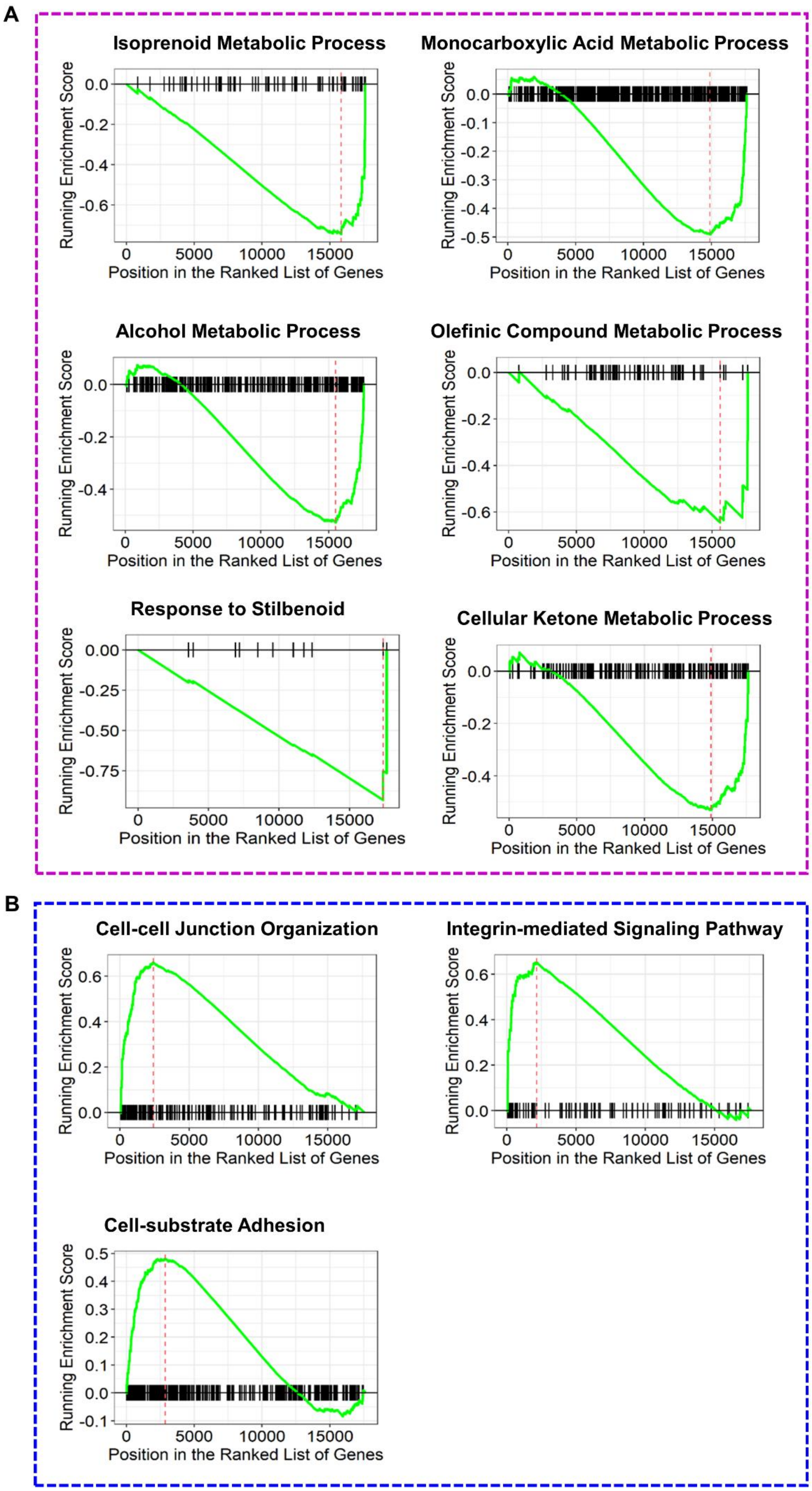
A
Isoprenoid Metabolic Process
Monocarboxylic Acid Metabolic Process
Alcohol Metabolic Process
Olefinic Compound Metabolic Process
Response to Stilbenoid
Cellular Ketone Metabolic Process
B
Cell-cell Junction Organization
Integrin-mediated Signaling Pathway
Cell-substrate Adhesion
Running Enrichment Score
Position in the Ranked List of Genes
0
5000
10000
15000

Figure S2. Maternal obesity induces impairments to E9.5 embryos.
(A). & (B). Representative terms of biological processes that are enriched by gene set enrichment analysis (GSEA) on the whole cell population in the MO group.

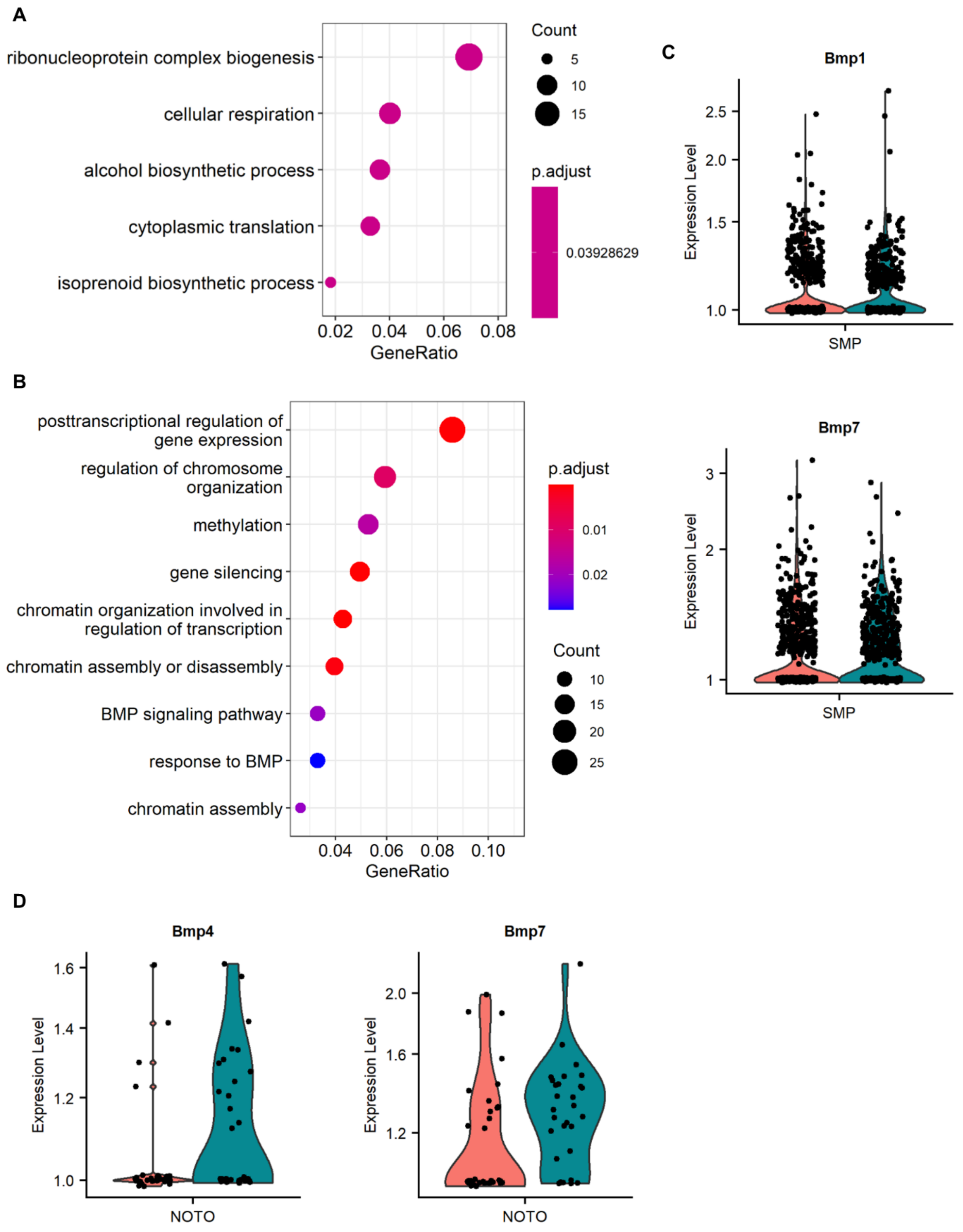

Figure S3. Maternal obesity induces impairments to the dermomyotomal cells of E9.5 embryos.

(A). Terms of biological processes that are enriched by Gene Ontology (GO) term analysis on down-regulated genes of dermomyotomal cells in the MO group.

(B). Terms of biological processes that are enriched by GO term analysis on up-regulated genes of dermomyotomal cells in the MO group.

(C-D). Expression of BMP ligands in the dermomyotome surrounding tissues. SMP, somatopleure; NOTO, notochord.
For (A)-(B), the *P*.adj (Benjamini-Hochberg adjusted *P* value) < 0.05; false discovery rate (FDR) (*q* value) < 0.05.

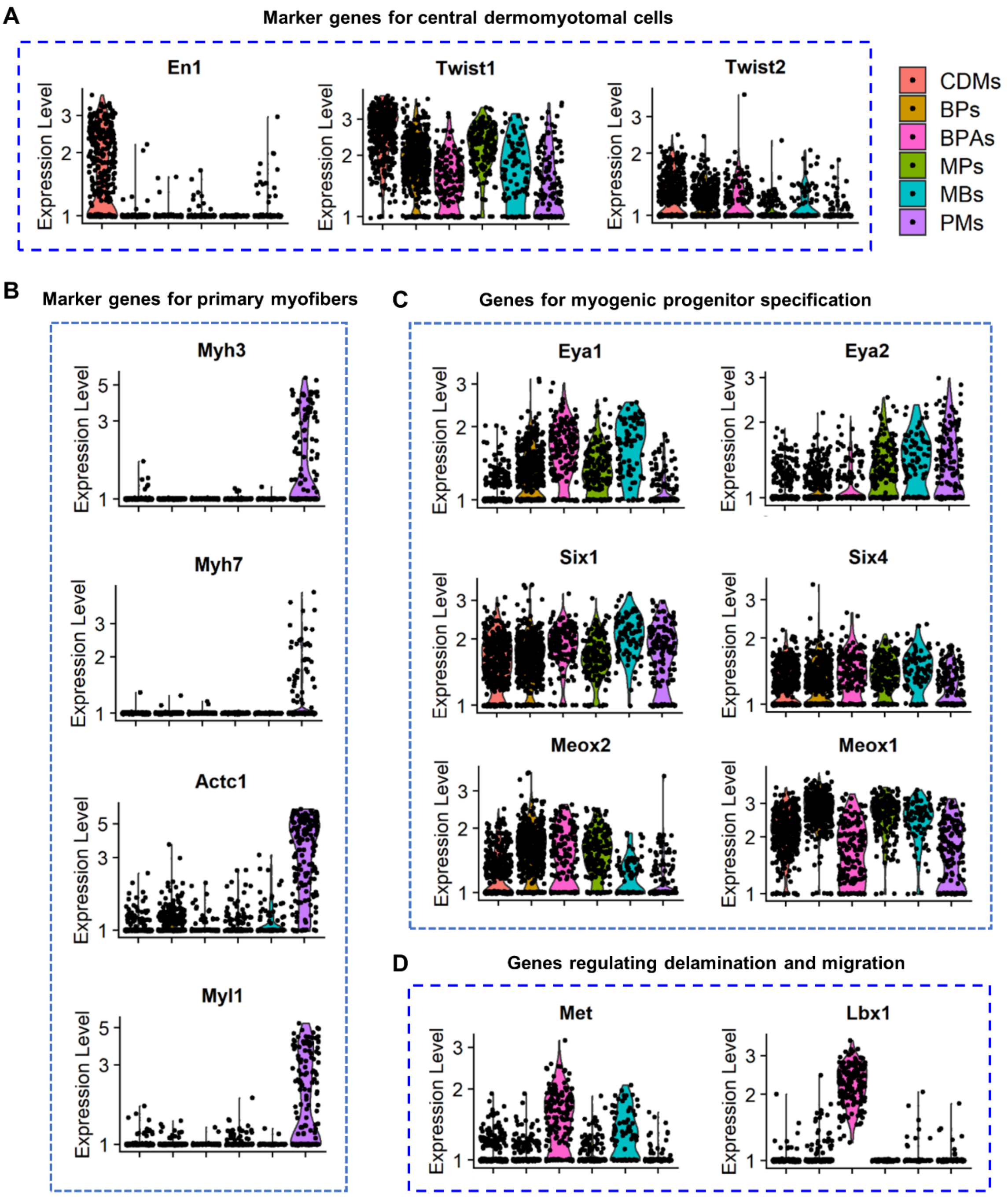

Figure S4. VlnPlot showing the expression of marker genes across different cell populations within the dermomyotome.

(A). Marker genes for central dermomyotomal cells.

(B). Gene markers for mature primary myofibers.

(C). Genes with regulatory functions for myogenic progenitor specification.

(D). Genes regulating delamination and migration of early progenitors.

CDMs, central dermomyotomal cells; MPs, myogenic progenitors; MBs, myoblasts; PMs, primary myofibers; BPs, brown adipogenic progenitors; BPAs, brown preadipocytes.

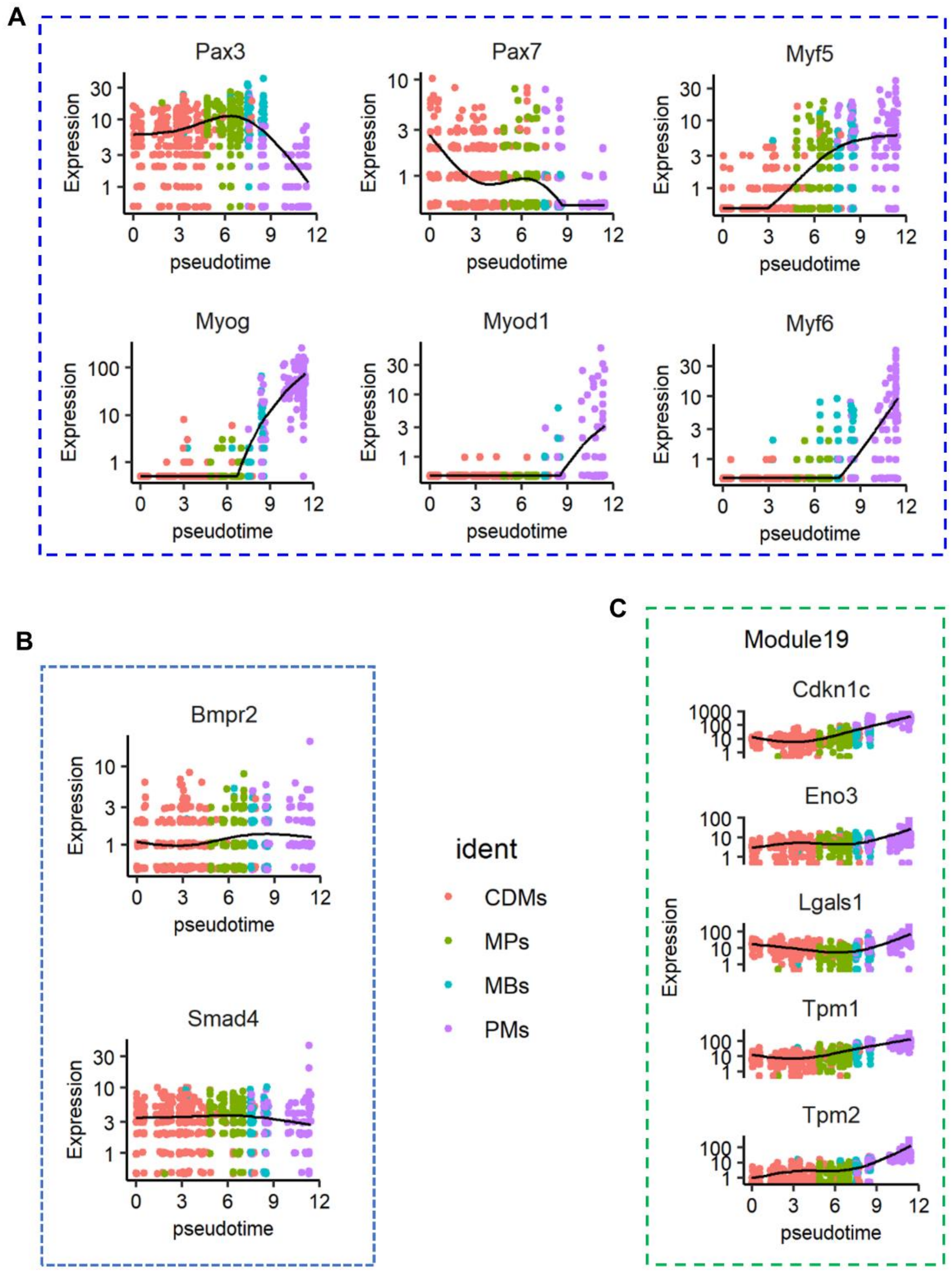

Figure S5. Pseudotemporal changes of representative genes across the pseudo-myogenic trajectory.

(A). Myogenic regulatory factors.

(B). BMP signaling related factors.

(C). Representative gene expression detected in Module 19.

CDMs, central dermomyotomal cells; MPs, myogenic progenitors; MBs, myoblasts; PMs, primary myofibers.

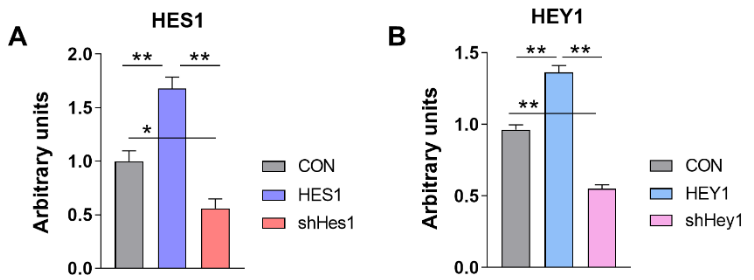

Figure S6 Arbitrary units of HES1 and HEY1 for immunoblotting in Figure 6D and Figure 6G, respectively.

**P < 0.01, *P < 0.05, mean ± SEM; N = 3 (one-way AVOVA followed by Bonferroni correction).

Table Captions for Table S1-13

Table S1. Gene expression profile for each cluster identified in E9.5 embryos

Table S2. Enriched GO terms of biological processes on down-regulated genes in the MO group of E9.5 embryos

Table S3. Enriched GO terms of biological processes on up-regulated genes in the MO group of E9.5 embryos

Table S4. Enriched GSEA terms of biological processes in the MO group of E9.5 embryos

Table S5. Enriched GSEA terms of biological processes in the dermomyotome of the MO group

Table S6. Enriched GO terms of biological processes on down-regulated genes in the dermomyotome of the MO group

Table S7. Enriched GO terms of biological processes on up-regulated genes in the dermomyotome of the MO group

Table S8. Gene expression profiles for sub-populations identified within the dermomyotome

Table S9. Genes classified in Module 16 of the pseudotemporal model (Figure 5E)

Table S10. Genes classified in Module 19 of the pseudotemporal model (Figure 5E)

Table S11. Enriched GSEA terms of biological processes in the myogenic progenitors

Table S12. Enriched GSEA terms of biological processes in the myoblasts

Table S13. Enriched GSEA terms of biological processes in the primary myofibers

Table S14. Primer sequences used for real-time quantitative PCR (qPCR)

Gene	Forward (5′–3′)	Reverse (5′–3′)	Access No.
18s	TTGTACACACCGCCCGTCGC	CTTCTCAGCGCTCCGCCAGG	NR_003278
HIF1a	AGGATGAGTTCTGAACGTCGAAA	CTGTCTAGACCACCGGCATC	NM_001313919
Hes1	GTCTACCTCTCTCCTTGGTCCT	AGGCCGTCTTTGGTTTGTCC	NM_008235
Hey1	CGTGAGTGGGATCAGTGTGC	CTCGATGATGCCTCTCCGTC	NM_010423
Mef2c	AGCACTGACAAAGGTCTGGT	AGGAAGTTGTTCCCGTCAGC	NM_001170537
Mef2a	TTGGAATGAACAGTCGGAAAC	CTAGTCCCTGTGGAGGCAAG	NM_001033713
Myog	GAGATCCTGCGCAGCGCCAT	CCCCGCCTCTGTAGCGGAGA	NM_031189
Bmp1	TCTTGGAGCGCACAGATGAG	TATACTCCTGCCCTGGCTGT	NM_001360021
Bmp2	TTTGGCCTGAAGCAGAGACC	ACGGCTTCTTCGTGATGGAA	NM_007553
Bmp4	GCAGGAACCAATGAGACACC	TCTTCCCGGTCTCAGGTATCA	NM_001316360
Bmp7	GGAGTAATCGCAAGCCTCGT	ACAGTGGCTTCTGCTTGGTT	NM_007557

Table S15. Primer sequences used for ChIP-qPCR

Gene	Forward (5′–3′)	Reverse (5′–3′)	Size (bp)
HES1 NG CON	TGGCACCCGTATCACAAAGT	GGCTCCACTTGGGAGTCTAAC	114
HEY1 NG CON	AAGGCTTGGTGTGGCATCAT	GTGGCCCAAGGGTTAAATGC	116
HES1 PS CON	TGTTTATTCAGCCGGGAGTC	CTCCTTGCAAACTCTCCATTC	83
HEY1 PS CON	CTGAGGCAGATCCTGTGTGA	CACCCACTCACTCCCAGACT	135
Mef2c Promoter A -727/-607	CATGACTGCCAAAGTGGAG	GATAGGCAGGCGGTATTCATTG	120
Mef2c Promoter B -263/-100	GGCAAATAACTACAGTGCT	TCCTCATTTCACACAGGCT	164
Mef2c Promoter C +42/+172	GCACTGACAAAGGTCTGGTTGT	TTGATCGTCAAGCGGTGAGATG	131

Footnote: HES1 NG CON, Hes1 flanking region was taken as negative control for HES1-bound sites. HEY1 NG CON, Hey1 flanking region was taken as negative control for HEY1-bound sites. HES1 PS CON, Mash1/Ascl1 promoter region was taken as the positive control for HES1-bound sites. HEY1 PS CON, Foxc1 promoter region was taken as the positive control for HEY1-bound sites.
